# Effectiveness of a facebook-delivered physical activity intervention for post-partum women: a randomized controlled trial protocol

**DOI:** 10.1186/1471-2458-13-518

**Published:** 2013-05-29

**Authors:** Jocelyn Kernot, Tim Olds, Lucy K Lewis, Carol Maher

**Affiliations:** 1 Health and Use of Time (HUT) group, Sansom Institute of Health Research, School of Health Sciences, University of South Australia, Adelaide, Australia

**Keywords:** Protocol, Randomized controlled trial, Facebook, Physical activity, Post-partum

## Abstract

**Background:**

Physical activity is reduced during the post-partum period. Facebook is frequently used by Australian mothers, and offers flexibility, high levels of engagement and the ability to disseminate information and advice via social contacts. The Mums Step it Up Program is a newly developed 50 day team-based physical activity intervention delivered via a Facebook app. The program involves post-partum women working in teams of 4–8 friends aiming to achieve 10,000 steps per day measured by a pedometer. Women are encouraged to use the app to log their daily steps and undertake social and supportive interactions with their friends and other participants. This study aims to determine the effectiveness of the Mums Step it Up Program.

**Method/design:**

A sample of 126 women up to 12 months post-partum will be recruited through community-based health and family services. Participants will be randomly allocated into one of three groups: control, pedometer only and the Mums Step it Up Program. Assessments will be completed at baseline, 6 weeks and 6 months. The primary outcome (objective physical activity) and the secondary outcomes (sleep quality and quantity, depressive symptoms, weight and quality of life) will be used to determine the effectiveness of the Mums Step it Up Program compared with the control and pedometer only groups. Analyses will be undertaken on an intention-to-treat-basis using random effects mixed modeling. The effect of theorized mediators (physical activity attitudes, subjective norms and perceived behavioral control) will also be examined.

**Discussion:**

This study will provide information about the potential of a Facebook app for the delivery of health behavior interventions. If this intervention proves to be effective it will be released on a mass scale and promoted to the general public.

**Trial registration:**

Australia and New Zealand Clinical Trials Register: ACTRN12613000069752

## Background

In Australia, inactivity is one of the most prevalent modifiable health behaviors impacting on the financial burden of the health care system [1]. Policies and initiatives to increase physical activity are a major focus for national governments [1,2]. Globally, physical inactivity is attributed with the cause of six per cent of deaths (this is equal to high blood glucose 6%, and behind high blood pressure 13%, and smoking 9%) [3].

Physical activity levels decline during early adulthood for both sexes, with women having greater levels of physical inactivity than men [1,4]. This decline has been attributed, in part, to life changes. For women, life events such as getting married and having a baby, lead to greater inactivity when compared to women who have not experienced these events [5,6]. Periods of life transition have been highlighted as key areas for future research and for targeted physical activity interventions [5,6]. Focusing specifically on the post-partum period (12 months following giving birth), physical inactivity has been linked with perceived lack of time, long working hours, lack of child care and the number of children in the home [6,7].

Health problems (physical and psychological) are common post-partum and can often interfere with daily activities [8,9]. Common issues include backache, headaches, anxiety, fatigue, sleep disturbance (above and beyond waking directly related to the baby), depression and decreased libido [8]. The post-partum period has been identified as a “potentially critical period for the development of obesity” [6], p. 312] with weight gain and weight retention common. Poor maternal physical and psychological health has been found to impact on the health of children, leading to possible issues with behavior, general physical health [9] and cognitive development [10]. Furthermore if women enter subsequent pregnancies in poor health (i.e. due to obesity and other health issues) they can expose their baby to negative health outcomes [11].

Regular physical activity has been associated with numerous direct health benefits for post-partum women including favorable effects on cholesterol levels, insulin sensitivity, and aerobic fitness [12] and increased chance of returning to pre-pregnancy weight [13,14]. Psychological benefits include a reduction in symptoms of anxiety and depression and improved mood and wellbeing [15].

To maximize the impact of health interventions, researchers are starting to see the value of targeting social networks. In recent years there has been increasing recognition that health problems such as obesity “spread” among social groups, and these effects are not limited to close friends and families, but also friends of friends [16,17]. This spread is possibly due to shared nutritional and lifestyle habits, and environmental factors [18]. Intervention programs may be able to exploit these social influences to promote positive health behavior [18].

Social networking websites such as Facebook provide enormous potential for the delivery of public health intervention programs [19] and offer the opportunity to target individuals and their social groups. While numerous interventions have been developed using stand-alone websites, a social networking-based intervention delivered via Facebook may offer additional advantages. Users visit Facebook frequently, increasing potential exposure to the intervention [20] which may assist with overcoming challenges of retention and adherence [21,22]. The primary function of Facebook is to facilitate communication, therefore it may assist with promoting interconnectivity and support among participants and can be utilized to target social networks to disseminate the intervention [20]. Facebook appeals to a wide demographic and its influence and usage in Australia and worldwide is likely to continue to grow [19].

To date there is a paucity of studies which have used Facebook to deliver health behavior change interventions. Examples are emerging which have used Facebook to encourage users to visit external intervention websites (e.g. sometimes referred to as a “fan” page, with a link to a standalone website), or to offer a discussion page, as part of a package of other intervention resources, which may also include text messaging, smart phone apps, and stand-alone websites [23,24].

Bartholomew et al. 2012 [25] examined new parents’ use of Facebook, and found that women’s Facebook use increased during their transition to parenthood and that a majority of women visited the site daily. These findings suggest that utilizing social networking websites for the delivery of interventions promoting positive health related behavior would be highly relevant to this population.

This paper outlines the protocol for a randomized controlled trial, designed to test the effectiveness of an online social networking physical activity intervention (Mum Step it Up Program) for post-partum women. The intervention in this study will be delivered entirely via a Facebook application (app).

## Methods/design

### Ethics

This study has been approved by the University of South Australia Human Research Ethics Committee (protocol number: 0000030420) and registered with the Australian New Zealand Clinical Trial Registry (registration number: ACTRN12613000069752).

### Aims

The aims of this study are to:

1) Determine the effectiveness of the Mums Step it Up Program for a) increasing physical activity and b) improving wellbeing, weight, sleep quality and quantity, and depressive symptoms.

2) Evaluate the possible role of Theory of Planned Behavior constructs (exercise attitudes, subjective norms and perceived behavioral control) in mediating behavior change.

3) Evaluate the feasibility and engagement of the Mums Step it Up Program.

### Intervention

The Mums Step it Up Program is a team-based 50-day intervention which aims to assist post-partum women to meet the well accepted adult health promotion recommendation of 10,000 steps per day [26]. Participants will work in teams of existing friends to reach the cumulative goal of half a million steps. Step counts will be measured daily using a pedometer. The program will be delivered via a Facebook app which has been developed based on the Theory of Planned Behavior [27,28] and Fun Theory [29]. The app has been designed to: be appealing and relevant to post-partum women; offer peer encouragement and support; provide short term and long term goals; and to be fun. The emphasis is on the health benefits of physical activity which may include: increased energy levels, and improved mood, well-being, and sleep.

The app will be used during both the recruitment process and the intervention phase. Team leaders (‘captains’) will be recruited via community based health and family services and word of mouth and asked to recruit 3–7 of their eligible Facebook friends to become team members. During the intervention phase participants will use the app to: record their daily step counts on a calendar (steps can be recorded up to 7 days in arrears); monitor their own progress compared to that of their team mates on a tally board; compare their team’s progress to that of other teams at a similar stage of the program (represented graphically); and interact with others (i.e. both team mates and participants outside their team) using the message walls. The message walls and other interactive features of the app allow for brief informal social interaction between participants.

The app provides further feedback regarding individual step achievements by detailing uncompensated (i.e. not taking into account other lifestyle factors such as diet) statistics on fat burned, hours of life gained, and carbon emissions and transport costs saved. In addition, the team captain also is encouraged to play a role in monitoring and supporting their team members. The app assists the captain in this, by providing information about each team member’s step logging behavior, total number of steps logged, tips for encouragement and a reminder to organize a chaser event (such as a night out) to celebrate the team’s achievements at the end of the 50 day challenge. All participants will also receive a weekly email summarizing their progress and reminding them to log onto the app to record their steps.

**Figure 1:**
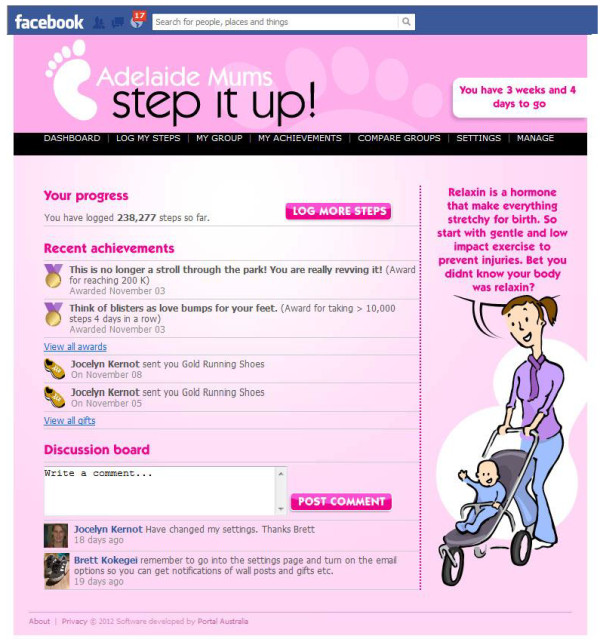
Screen shot of the Mums step it up Facebook app.

The app includes a number of features which have been specifically designed to add an element of fun and to maximize social interaction. A comedian has been consulted to assist with making these features light-hearted and comical. These include: daily tips for increasing physical activity; awards for individual and team step logging and step count achievements, which can be unlocked as participants progress through the challenge (based on the gaming industry); team members can also send virtual gifts to each other to congratulate others on their achievements (e.g. gold running shoes), or to provide encouragement and support (e.g. flowers) (Refer to Figure 1 for a screen shot of the Mums Step it Up app dashboard or home page).

### Recruitment and randomization

To be eligible women must be: 1) up to 12 months post-partum, whether it is their first or subsequent child; 2) current Facebook users; 3) able to read and understand English; 4) and live in greater metropolitan Adelaide. Women should not have a medical condition that would prevent them from participating in a walking program or be pregnant, or planning to fall pregnant in the next three months. Recruitment will occur on a rolling basis over a 6–12 month period. The team forming process occurs in two stages. *Stage one* involves recruitment of team captains, via advertising flyers in community-based health and family services around greater metropolitan Adelaide. Potential participants who respond to the flyer will be directed to the Mums Step it Up Facebook app, where they will view an online information video and complete a brief online form to confirm their eligibility, at which point they will become a “team captain”. They will then use the Facebook app to invite eligible friends from their existing Facebook network to join their team (*Stage two* of team formation). The Facebook app will send invitations to the nominated potential team members inviting them to join their friend’s (the team captain’s) team. These women will view an informative video, register their details and provide preliminary consent to participate in the study.

Team captains receive daily emails informing them of their friends’ responses to the invitations. Once at least 3 friends have joined the team, the team captain uses the app to finalize their team. At this point, all participants in the team will be contacted to arrange a face to face appointment with the Principal Investigator where full informed consent will be gained and baseline assessments completed. Once this assessment has been completed for all members of a team, the team will be randomly allocated (using block randomization) to one of three conditions – 1) Mums Step it Up Program, 2) pedometer only, or 3) control. The control group will receive basic written advice about the public health recommended guidelines of 10,000 steps per day and tips on how to achieve these. They will also be placed on a waiting list and provided with access to the Mums Step it Up Program on completion of the study. The pedometer only group will receive the same written advice as the control group as well as a pedometer and a log book to allow them to self-monitor their daily steps (for a period of 50 days). The intervention group will be given a pedometer and access to the full Mums Step it Up app.

### Power analysis

Based on a three group study with three repeated measures, ability to detect small to medium effect size differences (Cohen’s f = 0.25, Cohen’s d = 0.5), and an alpha of 0.05, 80% power will be achieved with a total sample size of 108. A design effect has been added to allow for clustering at the team level. Assuming teams of approximately six members, the design effect of this clustering is 1 + 0.01(6–1) = 1.05. Therefore, a total of 114 participants are needed for the study (108 × 1.05). This corresponds to 19 clusters of six participants, however to allow for an even number of clusters in all three groups (21 clusters with 6 participants) a total sample size of 126 will be recruited.

### Procedure

**Figure 2:**
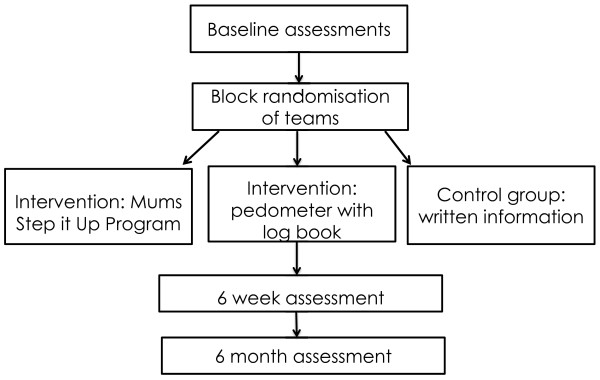
Overview of protocol procedure.

There will be three assessment points for the intervention and control groups: 1) baseline, 2) 8 weeks (coinciding with week six of the intervention), and 3) six months post baseline. Assessments one and three will occur at the participant’s home or at the University of South Australia, at the participants’ discretion. Assessment two will be undertaken remotely, with instruments posted to participants in a reply paid envelope. An overview of the protocol procedure is provided in Figure 2.

### Outcome measures

The primary outcome will be objective physical activity and the secondary outcomes will be sleep quality and quantity, mood, weight and wellbeing. The psychometric properties of the outcome measures are provided in Table 1. This study will also evaluate the possible role of Theory of Planned Behavior constructs (exercise attitudes, subjective norms and perceived behavioral control) in mediating behavior change.

**Table 1:** Psychometric properties of the outcome measures in this study

**Instrument**	**Reliability**	**Validity**
Edinburgh Postnatal Depression Scale (EPDS)	Not reported	Scale had **100% sensitivity** (percentage of true cases identified) and **89% specificity** (percentage of true non cases identified as such) when tested against the DSMIIIR diagnostic criteria for major depression (for Australian sample of 103 postpartum women) [30].
General Sleep Disturbance Scale (GSDS)	Internal consistency reliability coefficient of **.85**[31,32].	Validated with sleep logs **(r = .41)** and Actigraph results **(r = −.42)**[31]
Assessment of Quality of Life 8D (AQoL8D)	Test retest reliability of **.91** (baseline and 2 weeks) and **.89** (baseline and one month) [33]	When compared with 6 other Quality of Life Instruments* the AQoL8D had an average intra class correlation coefficient of **.57**[34].
Actigraph Accelerometer	Overall intra- and inter- instrument reliability for activity counts: **mean CV intra = 2.9%** and **mean CV inter = 3.5%**[35]	Validated with doubly labeled water **(r = 0.58, p < 0.001)**[36]

The 6 Quality of life instruments that the AQOL8D was compared with included: EuroQoL, Health Utilities Index Mark 3, Short Form Six Dimensions, Person Wellbeing Index, Satisfaction with Life Scale and Kessler Psychological Distress Scale.

#### Primary outcome

**Physical activity** Objective physical activity will be assessed using triaxial accelerometers (Model: Actigraph GTX3+). A five second epoch will be used with a sample rate of 80 Hz. Participants will be required to wear the accelerometer on their right hip in line with the mid-axillary line, 24 hours per day, for 7 days (except during water activities). A log sheet will be kept to record sleep time. Participants will be fitted with an accelerometer at baseline, and written information will be provided about the wearing regime. A reply-paid envelope will be provided for accelerometers and log sheets to be returned (after 7 days). To meet compliance thresholds participants will need to wear the accelerometer for 12 hours per day for four of the 7 days (including one weekend day) for data to be included. Ninety consecutive zeros (90 minutes) will be used to indicate non-wear time.

#### Secondary outcomes

**Sleep quality and quantity** Sleep quality and quantity will be evaluated with the General Sleep Disturbance Scale (GSDS), a self- rated questionnaire which evaluates sleep patterns over the past week and has been used previously with post-partum women [31]. The seven GSDS sub-scales include: “difficulty getting to sleep (1 item), waking up during sleep (1 item), waking up too early from sleep (1 item), quality of sleep (3 items), quantity of sleep (2 items), daytime sleepiness (7 items) and use of substances to help induce sleep (6 items)” [31], p. 112]. In total there are 21 items all of which are rated on an eight point scale, from zero (“never”) to seven (“every day”), the higher the score the greater the person’s sleep disturbance [31].

**Depressive symptoms** Depressive symptoms will be evaluated using the Edinburgh Postnatal Depression Scale (EDPS) [37]. The EPDS is a self-rated questionnaire consisting of 10 items, which takes approximately five minutes to complete. The 10 items consist of statements relating to the following depressive symptoms: inability to experience pleasure, self-blame, anxiety, fear or panic, inability to cope, difficulty sleeping, sadness, tearfulness and self-harm ideas [38]. Women are required to read the statements and indicate the response which most applies to how they have been feeling over the last seven days. Scores range from 0–3 for each item and can be summed at the completion of the questionnaire to establish a total score [37,39]. The higher the score, the greater the risks of postnatal depression, the maximum score that can be obtained is 30. A score of 10 or greater may be indicative of possible postnatal depression [40].

**Quality of life** Participants’ wellbeing and quality of life will be assessed using the Assessment of Quality of Life (AQoL) instrument [41], which measures health-related quality of life. The AQoL addresses a number of health- related domains relevant to post-partum women including: independent living, relationships, mental health, pain, senses, self-worth and happiness. This is a self-rated questionnaire which has a total of 35 items. Each item has a statement (with an option of 4–6 responses depending on the item); participants are required to mark the response which is most representative of their situation [42]. The developers of the AQOL questionnaire have produced an algorithm which combines responses into dimension scores and a single utility score. The questionnaire can also be scored without utility weights [42]. Scores can be compared with normative data for the general Australian population.

**Weight** Weight will be determined through height (Victa Stadiometer) and weight (Tanita HD332 electronic scales) measurements taken according to International Society for the Advancement of Kinanthropometry protocols [43], and calculated according to the criteria of the World Health Organization [44].

#### Theorized mediators

**Theory of planned behavior questionnaire** This self-administered questionnaire will be used to assess possible mediators of behavior change. The questionnaire has been designed specifically for this study and is based on Francis et al. [45] and Ajzen [46] guidelines for developing a Theory of Planned Behavior questionnaire. The questionnaire will measure the mediator variables of attitude, subjective norms and perceived behavioral control. These variables will be included as subscales. Each item includes a seven point scale and participants will be asked to circle the number that best describes their opinion. The mean for all items on a subscale will be calculated. The higher the persons mean score, for each subscale, the greater the possible influence of the mediator on behavioral change. A copy of the questionnaire has been provided in an Additional file 1.

### Statistical analysis

The intention-to-treat principle will be used for data analysis whereby all participants randomized at the commencement of the trial will remain in the sample for analysis [47]. Random effect mixed modeling will be used to determine the effectiveness of the intervention program. The relationship between primary and secondary outcomes and demographic variables will be assessed and where relationships exist the demographic variables will be used as covariates.

### Process evaluation

The process evaluation will occur concurrently with the randomized controlled trial and will assess the feasibility (i.e. whether the program is suitable for postpartum women) and engagement (how much the participants use the app) of the Mums Step it Up program.

**Feasibility:** will be assessed on the basis of participants’ recall, use and satisfaction with the program, using self-report items developed and used previously by the investigators. A copy of the feasibility instrument has been provided in Additional file 2.

**Engagement:** will be assessed on the basis of login statistics. Specifically, the number and duration of logins, frequency of logging daily steps and number of interactions with team members will be recorded.

Feasibility and engagement data will be descriptively analyzed. In addition, sub-group analysis will be undertaken to determine whether the intervention effectiveness is related to dosage and/or compliance.

## Discussion

Increased physical activity provides both physical and psychological health benefits for post-partum women [12-15]; however flexible programs are required to meet the specific lifestyle needs of this population [15,48]. This study will evaluate the effectiveness of a social networking physical activity intervention which has been designed to specifically address the lifestyle requirements of post-partum women. Facebook will be used as the intervention platform as post-partum women are frequent Facebook users [25] and participants will be able to log on at a convenient time. It is anticipated that the social/team nature of the intervention will enhance effectiveness by providing accountability, influencing expectations, and offering support and encouragement. The Mums Step it Up program has been designed to be fun to increase the likelihood that women will want to interact and engage with the app on a regular basis.

Research using Facebook to promote positive behavioral change has been limited. Facebook is ingrained in the lifestyles of millions people worldwide [49], and therefore may offer mass-reach, appeal, accessibility, saliency and sustainability. Should the results of this study be affirmative, the app will be refined and disseminated on a mass scale potentially benefiting large numbers of post-partum women in Australia and internationally. The program may also be adapted and utilized to promote positive health behavior with other population groups such as those living in rural and remote communities, teenage girls, or people living with diabetes mellitus.

## Competing interests

The authors declare that they have no competing interests that are directly related to the content of this manuscript.

## Authors’ contributions

All authors have contributed to the protocol design and revised and edited the manuscript. CM and TO conceived the idea of the study. CM conceived the idea of the Mums Step it Up Facebook app and has led the development and content of the app. JK has contributed to the Mums Step it Up Facebook app content. JK drafted the manuscript. All authors read and approved the final manuscript.

## Pre-publication history

The pre-publication history for this paper can be accessed here:

http://www.biomedcentral.com/1471-2458/13/518/prepub

## Supplementary Material

Additional file 1 Theory of Planned Behavior Questionnaire; Copy provided of the Theory of Planned Behavior Questionnaire that will be used in this study. Click here for file

Additional file 2 Feedback Questionnaire (for Process Evaluation); Copy provided of the Feedback Questionnaire that will be used in the Process Evaluation. Click here for file
