# Supplementary material for: Effectiveness of a facebook-delivered physical activity intervention for post-partum women: a randomized controlled trial protocol
Source: BMC Public Health. 2013 May 29;13:518. doi: 10.1186/1471-2458-13-518 (PMC3674954; doi:10.1186/1471-2458-13-518)
Supplement: Additional file 1 — Theory of Planned Behavior Questionnaire; Copy provided of the Theory of Planned Behavior Questionnaire that will be used in this study. [file 1471-2458-13-518-S1.docx]

**Theory of Planned Behaviour Questionnaire**

Thank you for agreeing to participate in the research study titled: “The Mums Step it Up Program- a social networking physical activity intervention for post-partum women”. We would like to find out some information about how you feel about exercising. As you are aware this research study aims to assist women who have recently had a baby to achieve 10,000 steps per day, therefore the questions below relate to this.

Please complete the questions below by circling the number that most applies to how you feel. If you feel uncomfortable about answering any of the questions please leave them blank

**For me, achieving 10,000 steps per day over the next month would be:**

harmful :___1__:___2__:___3__:___4__:___5__:___6__:___7___: beneficial

useless :___1__:___2__:___3__:___4__:___5__:___6__:___7___: useful

unimportant :___1__:___2__:___3__:___4__:___5__:___6__:___7___: important

unenjoyable :___1__:___2__:___3__:___4__:___5__:___6__:___7___: enjoyable

boring :___1__:___2__:___3__:___4__:___5__:___6__:___7___: fun

painful :___1__:___2__:___3__:___4__:___5__:___6__:___7___: pleasurable

**I think that if I were to achieve 10,000 steps per day over the next month, most people who are important to me would be:**

disapproving :___1__:___2__:___3__:___4__:___5__:___6__:___7___: approving

unsupportive :___1__:___2__:___3__:___4__:___5__:___6__:___7___: supportive

discouraging :___1__:___2__:___3__:___4__:___5__:___6__:___7___: encouraging

**Most people who are important to me, will themselves exercise regularly over the next month**

disagree :___1__:___2__:___3__:___4__:___5__:___6__:___7___: agree

**Over the next month, most people who are important to me will be physically:**

inactive :___1__:___2__:___3__:___4__:___5__:___6__:___7___: active

**Over the next month, the exercise levels of most people who are important to me will be:**

low:___1__:___2__:___3__:___4__:___5__:___6__:___7___: high

**Achieving 10,000 steps per day, for the next month, would be:**

possible :___1__:___2__:___3__:___4__:___5__:___6__:___7___: impossible

**I would have complete control over whether or not I achieved 10,000 steps per day, for the next month.**

untrue :___1__:___2__:___3__:___4__:___5__:___6__:___7___: true

**Achieving 10,000 steps per day over the next month would be completely up to me**

disagree:___1__:___2__:___3__:___4__:___5__:___6__:___7___: agree

**Achieving 10,000 steps per day over the next month would be:**

difficult:___1__:___2__:___3__:___4__:___5__:___6__:___7___: easy

**I am confident that I can achieve 10,000 steps per day, for the next month**

unconfident:___1__:___2__:___3__:___4__:___5__:___6__:___7___: confident

**I am certain that I could achieve 10,000 steps per day, for the next month**

uncertain:___1__:___2__:___3__:___4__:___5__:___6__:___7___: certain

**I strongly intend to do everything I can, to achieve 10,000 steps per day for the next month**

untrue:___1__:___2__:___3__:___4__:___5__:___6__:___7___: true

**I am committed to achieving 10,000 steps per day over the next month.**

uncommitted:___1__:___2__:___3__:___4__:___5__:___6__:___7___: committed

**I am motivated to achieve 10,000 steps per day for the next month**

unmotivated:___1__:___2__:___3__:___4__:___5__:___6__:___7___: motivated
