# Supplementary material for: Effectiveness of a facebook-delivered physical activity intervention for post-partum women: a randomized controlled trial protocol
Source: BMC Public Health. 2013 May 29;13:518. doi: 10.1186/1471-2458-13-518 (PMC3674954; doi:10.1186/1471-2458-13-518)
Supplement: Additional file 2 — Feedback Questionnaire (for Process Evaluation); Copy provided of the Feedback Questionnaire that will be used in the Process Evaluation. [file 1471-2458-13-518-S2.docx]

**Feedback Questionnaire**

Thank you for agreeing to participate in the research study titled: “The Mums Step it Up Program- a social networking physical activity intervention for post-partum women”. We would like to get your feedback about the program.

Please complete the questions below by circling the number that most applies to how you feel. If you feel uncomfortable about answering any of the questions please leave them blank

**The Facebook app was easy to use:**

agree :___1__:___2__:___3__:___4__:___5__:disagree

comment: __________________________________________________________________ ___________________________________________________________________________

**The Facebook app is of interest to women who have recently had a baby.**

agree :___1__:___2__:___3__:___4__:___5__:disagree

comment: __________________________________________________________________

___________________________________________________________________________

**The language used in the Facebook app is easy to follow**

agree :___1__:___2__:___3__:___4__:___5__:disagree

comment: __________________________________________________________________

___________________________________________________________________________

**I enjoyed using the Facebook app.**

agree :___1__:___2__:___3__:___4__:___5__:disagree

comment: __________________________________________________________________

__________________________________________________________________________

**The look and features of the Facebook app are appealing.**

agree :___1__:___2__:___3__:___4__:___5__:disagree

comment: __________________________________________________________________

___________________________________________________________________________

**The selection of gifts available to send to team mates was sufficient.**

agree :___1__:___2__:___3__:___4__:___5__:disagree

comment: __________________________________________________________________

___________________________________________________________________________

**The awards were interesting and motivating.**

agree :___1__:___2__:___3__:___4__:___5__:disagree

comment: __________________________________________________________________

___________________________________________________________________________

**The statistics on life gained, fat burned, carbon saved and travel costs saved were interesting and motivating.**

agree :___1__:___2__:___3__:___4__:___5__:disagree

comment: __________________________________________________________________

___________________________________________________________________________

**The app provided me with enough feedback about the steps that I had accrued.**

agree :___1__:___2__:___3__:___4__:___5__:disagree

comment: __________________________________________________________________

___________________________________________________________________________

**The app allowed me to compare my progress, with my team mates.**

agree :___1__:___2__:___3__:___4__:___5__:disagree

comment: __________________________________________________________________

___________________________________________________________________________

**I would recommend this Facebook app to other women who have recently had a baby**

agree :___1__:___2__:___3__:___4__:___5__:disagree

comment: __________________________________________________________________

__________________________________________________________________________

**The Facebook app helped me to interact with my team members.**

agree :___1__:___2__:___3__:___4__:___5__:disagree

comment: __________________________________________________________________ ___________________________________________________________________________

**Logging my step counts on a daily basis was easy**

agree :___1__:___2__:___3__:___4__:___5__:disagree

comment: __________________________________________________________________ ___________________________________________________________________________

**The pedometer was easy to use**

agree :___1__:___2__:___3__:___4__:___5__:disagree

comment:_____________________________________________________________________________________________________________________________________________

**The Facebook app helped me to increase my physical activity**

agree :___1__:___2__:___3__:___4__:___5__:disagree

comment:_____________________________________________________________________________________________________________________________________________

**The Facebook app helped to promote competition**

agree :___1__:___2__:___3__:___4__:___5__:disagree

comment:_____________________________________________________________________________________________________________________________________________

**The pedometer helped me to increase my physical activity**

agree :___1__:___2__:___3__:___4__:___5__:disagree

comment:_____________________________________________________________________________________________________________________________________________

**My team mates were supportive**

agree :___1__:___2__:___3__:___4__:___5__:disagree

comment:_____________________________________________________________________________________________________________________________________________

**Being part of a team helped me to increase my physical activity**

agree :___1__:___2__:___3__:___4__:___5__:disagree

comment:_____________________________________________________________________________________________________________________________________________

What were the 3 best things about the Facebook app?


What were the 3 worst things about the Facebook app?


What changes would you suggest to help improve the Facebook app?
